# One-Stop Hybrid Coronary Revascularization Versus Off-Pump Coronary Artery Bypass Grafting in Patients With Multivessel Coronary Artery Disease

**DOI:** 10.3389/fcvm.2021.755797

**Published:** 2021-12-17

**Authors:** Dongjie Li, Yulin Guo, Yingdi Gao, Xiangguang An, Yan Liu, Song Gu, Xitao Zhang, Jiuchang Zhong, Jie Gao, Pixiong Su

**Affiliations:** ^1^Department of Cardiac Surgery, Heart Center and Beijing Key Laboratory of Hypertension, Beijing Chaoyang Hospital, Capital Medical University, Beijing, China; ^2^Heart Center and Beijing Key Laboratory of Hypertension, Beijing Chaoyang Hospital, Capital Medical University, Beijing, China

**Keywords:** hybrid coronary revascularization (HCR), off-pump coronary artery bypass graft (OPCAB), percutaneous coronary intervention (PCI), minimally invasive direct coronary artery bypass (MIDCAB), major adverse cardiovascular and cerebrovascular events (MACCE)

## Abstract

**Background:** Data on one-stop hybrid coronary revascularization (HCR) are limited. This study aimed to compare the early and midterm outcomes of one-stop HCR with off-pump coronary artery bypass grafting (OPCAB) in patients with multivessel coronary artery disease.

**Methods:** From April 2018 to May 2021, 752 patients with multivessel coronary artery disease who underwent isolated one-stop HCR or OPCAB were retrospectively included in this analysis. After exclusion and propensity score matching, 151 patients who underwent HCR were matched with 151 patients who underwent OPCAB. The primary endpoints were midterm major adverse cardiovascular and cerebrovascular events (MACCE) after the procedure. The secondary endpoints were in-hospital complications and outcomes.

**Results:** The preprocedural characteristics were well balanced between the two groups after matching. The HCR group was associated with a lower rate of perioperative transfusion (23.8 vs. 53.0%, *p* < 0.001) and new-onset atrial fibrillation (AF) (5.3 vs. 15.2%, *p* = 0.004), shorter time of mechanical ventilation (h) [15 (16, 17) vs. 17 (16, 20), *p* < 0.001], and shorter length of stay (LOS) in the hospital (days) [19 (16, 24) vs. 22 (18, 27), *p* = 0.001]. Cumulated MACCE rates were similar between the two groups (15.9 vs. 14.0%, *p* = 0.59) during a median follow-up of 20 months.

**Conclusions:** One-stop HCR is safe and efficacious with less invasiveness and faster postoperative recovery in selected patients with multivessel coronary artery disease. Randomized controlled trials with larger sample sizes and long-term follow-up are warranted to confirm these findings.

## Introduction

Coronary artery bypass grafting (CABG) remains the gold standard for the treatment of multivessel coronary artery disease (1–3). The left internal mammary artery (LIMA) to left anterior descending (LAD) graft provides most of the survival benefit of CABG due to its long-term patency rate, which can reach 90% at 10 years. However, the 10-year patency for saphenous vein graft (SVG) was only 60%, and conventional CABG is a relatively invasive and high-risk procedure via sternotomy (4–6). In-stent restenosis was <6% in patients undergoing percutaneous coronary intervention (PCI) with drug eluting stents (DESs) (7). However, the long-term outcomes of PCI were not superior to those of CABG in patients with intermediate or high SYNTAX scores (>22) (8, 9).

Hybrid coronary revascularization (HCR) was first introduced by Angelini in 1996 and consisted of LIMA-LAD anastomosis using a minimally invasive left thoracotomy approach and PCI procedure for non-LAD lesions (10). HCR combines the advantages of CABG and PCI, avoids their relative deficiencies, and achieves complete revascularization. The HYBRID trial and several observational studies have shown similar short and long-term outcomes compared with off-pump or on-pump CABG (11–14). Nevertheless, data comparing HCR and off-pump CABG (OPCAB) are still limited, and the safety and efficacy of HCR in multivessel coronary artery disease have not been completely indicated, especially for one-stop HCR patients, due to the majority composition of staged HCR in these studies. Considering the potentially different strengths and disadvantages between one-stop and staged HCR, the results of one-stop HCR need to be separately evaluated.

Therefore, we sought to investigate the early and midterm outcomes of one-stop HCR compared with OPCAB in patients with multivessel coronary artery disease to evaluate the safety and effectiveness of this procedure.

## Methods

### Study Population

**Figure 1:**
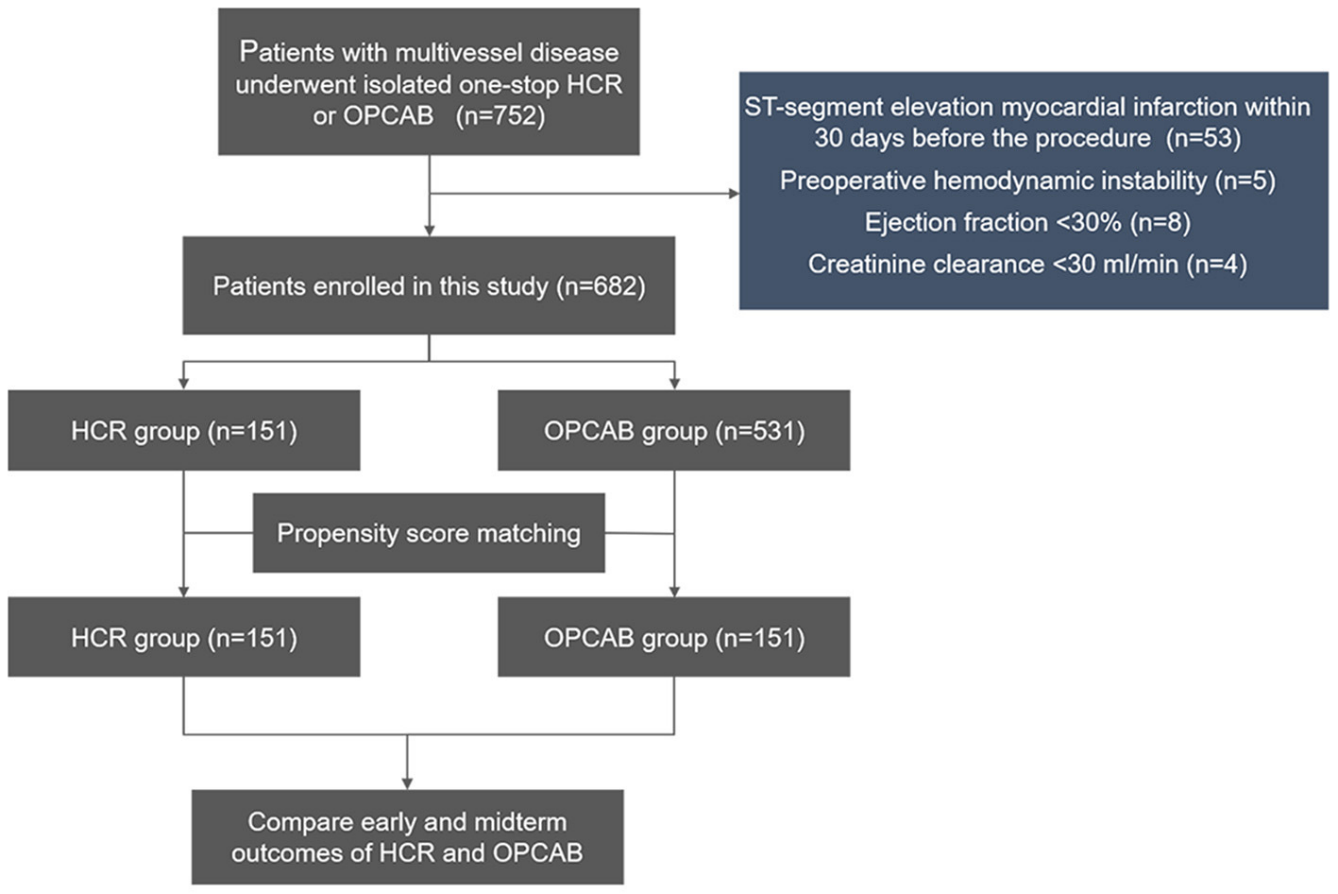
Flow chart of the study. HCR, hybrid coronary revascularization; OPCAB, off-pump coronary artery bypass graft.

This was a retrospective single-center observational study conducted at Beijing Chaoyang Hospital, Capital Medical University. From April 2018 to May 2021, data from a total of 752 patients with multivessel coronary artery disease who underwent isolated one-stop HCR or OPCAB were collected. Patients with ST-segment elevation myocardial infarction within 30 days before the procedure, ejection fraction <30%, hemodynamic instability, and creatinine clearance <30 ml/min were excluded. Finally, 151 patients underwent one-stop HCR (HCR group), and 531 patients who received OPCAB (OPCAB group) were enrolled in this study. Figure 1 shows the detailed flow of this study.

For the choice of revascularization strategies, all patients were reviewed and discussed preoperatively by the heart team of our center, which consisted of cardiac surgeons, interventional cardiologists, and anesthesiologists, to make the most appropriate decision regarding PCI, CABG, or HCR. The selection criteria of patients who underwent one-stop HCR were as follows: patients with multivessel disease in whom the LAD lesion was not suitable for PCI but was suitable for surgical revascularization and in whom the non-LAD lesions were amenable to PCI; and patients who were not good candidates for traditional CABG, such as poor right coronary or circumflex arteries for bypass, relative contraindication for sternotomy, porcelain aorta, lack of acceptable conduits, and patient desire for minimally invasive procedures.

### Data Collection

Preoperative risk profile and demographic features, including age, sex, body mass index (BMI), hypertension, hyperlipoidemia, diabetes mellitus (DM), and smoking status, were retrospectively extracted for all patients from the database of Chaoyang Hospital. Intraoperative and postoperative variables were also collected. The SYNTAX score and EuroSCORE II were calculated based on the anatomy of the lesions and preoperative risk factors. The study was conducted in accordance with the Declaration of Helsinki (as revised in 2013). The study was approved by the local research ethics board of Chaoyang Hospital (No.: 2021-D-5), and individual consent for this retrospective analysis was waived.

### Surgical Technique and Intervention

For the HCR group, all patients underwent one-stop HCR in the hybrid operating room. Surgical procedures were performed by minimally invasive direct coronary artery bypass (MIDCAB) or endoscopic assisted coronary artery bypass. LIMA was harvested as a pedicle directly through a small anterior thoracotomy (5–7 cm) at the fourth to fifth intercostal space using special retractors or via an endoscope to avoid chest wall retraction and rib spreading. Then, LIMA was hand sewn to the LAD territory via direct vision. All surgical procedures were performed by one experienced surgeon (Pixiong Su). A partial dose of protamine was administrated to neutralize heparin after LIMA-LAD anastomosis. Then, a loading dose of clopidogrel was administrated before closure of the thorax. The PCI procedures were performed according to practice guidelines and standard techniques (15). PCI for non-LAD lesions was performed through the femoral artery, and the femoral arterial sheath was placed before heparinization to avoid potential access site hematomas. The guidewire and stent selection were performed according to the interventionist's discretion. LIMA-LAD graft patency was immediately confirmed by angiography after chest closure. Then, DESs or drug-coated balloons (DCBs) were used to treat the non-LAD lesions.

For the OPCAB group, standard procedures described previously were followed (16). Aspirin was administered 100 mg daily after HCR and OPCAB procedures and then continued for life, while clopidogrel was administered at a dose of 75 mg/day for 12 months.

### Follow-Up

All patients needed to return to the outpatient department for a postoperative review at 1 and 6 months after discharge from the hospital and then once a year after surgery. Patients who did not return for review visits were contacted via telephone during the study period by the research staff using standard procedures and forms.

The primary endpoints of this study were midterm major adverse cardiovascular and cerebrovascular events (MACCE) after the procedure, including death, myocardial infarction (MI), stroke, and repeat revascularization (defined as any revascularization after the HCR procedure or isolated OPCAB procedure). The secondary endpoints were in-hospital complications and outcomes, defined as in-hospital death, MI, stroke, repeat revascularization, reoperation for bleeding, time of mechanical ventilation, mechanical ventilation (PMV), perioperative transfusion, renal failure requiring dialysis, new onset atrial fibrillation (AF), incision infection, intensive care unit (ICU) stay, and length of stay (LOS) in hospital (days).

### Statistical Analysis

To reduce the impact of selection bias and potential confounding factors in this observational study, propensity score matching was performed using a logistic regression model. We chose nearest-neighbor caliper matching without a replacement, and the matching ratio was 1:1. Key variables and risk factors were involved in the matching. The standardized differences (SD) were calculated to assess the balance for the baseline characteristics before and after matching. SD values <10% indicated good matching. All matching procedures were performed by R (version 4.0.3).

Continuous variables were expressed as the means ± standard deviation or medians (the 25th percentile and the 75th percentile), and categorical data were summarized as a proportion. Comparisons of baseline characteristics and outcomes between the HCR group and OPCAB group were assessed by *t test* or Mann–Whitney *U* test for continuous variables and chi-square test or Fisher exact test for categorical variables before and after matching. Kaplan–Meier curves and log-rank tests were performed to compare cumulative events and MACCE rates between the two groups after matching. All statistical data analyses were performed by SPSS (IBM Corp. Released 2013. IBM SPSS Statistics for Windows, Version 22.0. Armonk, NY: IBM Corp). *P* < 0.05 was considered statistically significant.

## Results

### Baseline Characteristics

Before propensity score matching, there were significant differences in demographics and comorbidities between the two groups. The HCR group had a higher BMI, a higher proportion of hyperlipidemia patients, administration of statins, and better heart function. Additionally, there were significantly lower EuroSCORE II scores, a lower proportion of diabetes mellitus patients, previous MI, and preoperative intra-aortic balloon pump (IABP) insertion compared with the OPCAB group. Variables of the unmatched population are shown in Table 1.

**Table 1:** Preoperative characteristics of unmatched patients who underwent hybrid coronary revascularization (HCR) and off-pump coronary artery bypass (OPCAB).

**Preoperative**	**HCR group**,	**OPCAB group**,	**SD**	***P***
**characteristics**	***N =* 151**	***N =* 531**		
Age (years)	64.6 ± 9.4	63.6 ± 8.8	0.051	0.58
Male	75.5	77.0	0.036	0.70
BMI (kg/m2)	26.0 ± 3.3	25.3 ± 3.2	0.189	0.04
Hypertension	72.8	68.9	0.085	0.35
Hyperlipidemia	64.9	54.0	0.218	0.02
Diabetes mellitus	37.1	46.1	0.187	0.048
Smoker	55	50.1	0.098	0.29
COPD	1.3	2.3	0.063	0.75
Peripheral vascular disease	9.9	6.6	0.135	0.16
Preoperative arrhythmia	7.9	9.2	0.044	0.63
Previous stroke	19.9	21.7	0.043	0.64
Previous MI	16.6	45.6	0.583	<0.001
Previous PCI	21.9	19.8	0.052	0.57
Acute coronary syndrome	98.7	96.2	0.128	0.19
Left main disease	41.7	36.9	0.100	0.28
LVEF (%)	63.6 ± 9.0	60.7 ± 11.0	0.265	0.001
LVEDD (mm)	48.0 ± 4.6	49.4 ± 5.8	0.235	0.003
Preoperative IABP	2.0	7.3	0.205	0.02
SYNTAX Score	30.1 ± 9.4	31.7 ± 8.0	0.195	0.07
EuroSCORE II	1.97 ± 1.67	2.93 ± 2.27	0.424	<0.001
β blocker	64.9	70.1	0.113	0.23
ACEI/ARB	40.4	35.2	0.109	0.24
Statin	96.0	86.8	0.272	0.002

*Values are presented as mean ± SD or %*.

*HCR, hybrid coronary revascularization; OPCAB, off pump coronary artery bypass grafting; SD, standardized difference; BMI, body mass index; COPD, chronic obstructive pulmonary disease; MI, myocardial infarction; PCI, percutaneous coronary intervention; LVEF, left ventricular ejection fraction; LVEDD, left ventricular end-diastolic dimension; IABP, intra-aortic balloon pump; ACEI, angiotensin converting enzyme inhibitor; ARB, angiotensin receptor blockers*.

There were 151 patients in each group after 1:1 propensity score matching, and the baseline characteristics were similar between the two groups (Table 2). In the HCR group, no patients required conversion to sternotomy or cardiopulmonary bypass (CPB), and the mean number of DESs or DCBs used in each patient was 2.3 ± 1.5. The LIMA-LAD anastomosis, mean graft flow (MGF), and pulsatility index (PI) were comparable between the two groups (Table 3).

**Table 2:** Preoperative characteristics of matched patients who underwent HCR and OPCAB.

**Preoperative**	**HCR group**,	**OPCAB group**,	**SD**	***P***
**characteristics**	***N =* 151**	***N =* 151**		
Age (years)	64.6 ± 9.4	64.4 ± 8.6	0.011	0.92
Male	75.5	77.5	0.047	0.68
BMI (kg/m^2^)	26.0 ± 3.3	25.3 ± 3.3	0.193	0.10
Hypertension	72.8	72.8	0.000	1.00
Hyperlipidemia	64.9	61.6	0.066	0.55
Diabetes mellitus	37.1	33.8	0.066	0.55
Smoker	55.0	51.7	0.066	0.56
COPD	1.3	2.0	0.045	1.00
Peripheral vascular disease	9.9	9.3	0.027	0.85
Preoperative arrhythmia	7.9	7.3	0.023	0.83
Previous stroke	19.9	19.9	0.000	1.00
Previous MI	16.6	16.6	0.000	1.00
Previous PCI	21.9	19.2	0.067	0.57
Acute coronary syndrome	98.7	98.7	0.000	1.00
Left main disease	41.7	43.7	0.041	0.73
LVEF (%)	63.6 ± 9.0	63.3 ± 9.2	0.021	0.83
LVEDD (mm)	48.0 ± 4.6	48.2 ± 5.4	0.028	0.78
Preoperative IABP	2.0	0.0	0.076	0.25
SYNTAX Score	30.1 ± 9.4	31.2 ± 7.4	0.081	0.51
EuroSCORE II	1.97 ± 1.67	2.36 ± 2.40	0.155	0.15
β blocker	64.9	65.6	0.015	0.90
ACEI/ARB	40.4	39.7	0.014	0.91
Statin	96.0	98.0	0.059	0.50

*Values are presented as mean ± SD or %*.

*CR, hybrid coronary revascularization; OPCAB, off pump coronary artery bypass grafting; SD, standardized difference; BMI, body mass index; COPD, chronic obstructive pulmonary disease; MI, myocardial infarction; PCI, percutaneous coronary intervention; LVEF, left ventricular ejection fraction; LVEDD, left ventricular end-diastolic dimension; IABP, intra-aortic balloon pump; ACEI, angiotensin converting enzyme inhibitor; ARB, angiotensin receptor blockers*.

**Table 3:** Intraoperative characteristics of matched patients who underwent HCR and OPCAB.

**Intraoperative**	**HCR group**,	**OPCAB group**,	***P***
**characteristics**	***N =* 151**	***N =* 151**	
Conversion to sternotomy	0	NA	NA
LIMA-LAD	98.7	95.4	0.17
MGF (ml/min)	23.5 ± 13.9	26.6 ± 19.7	0.11
PI	2.5 ± 1.4	2.4 ± 1.6	0.23
Number of DES/DCB	2.3 ± 1.5	NA	NA

*Values are presented as mean ± SD or %*.

*HCR, hybrid coronary revascularization; OPCAB, off pump coronary artery bypass grafting; LIMA, left internal mammary artery; LAD, left anterior descending artery; MGF, mean graft flow; PI, pulsatility index; DES, drug-eluting stents; DCB, drug-coated balloon*.

### In-Hospital Outcomes

The in-hospital outcomes are illustrated in Table 4. The incidences of in-hospital death, MI, stroke, and repeat revascularization were comparable between the two matched groups. Meanwhile, differences in the rate of reoperation for bleeding, PMV (≥48 h), renal failure requiring dialysis, incision infection, and length of ICU stay (h) were not statistically significant. The HCR group had a lower rate of perioperative transfusion (23.8 vs. 53.0%, *p* < 0.001) and new-onset AF (5.3 vs. 15.2%, *p* = 0.004), shorter time of mechanical ventilation (h) [15 (16, 17) vs. 17 (16, 20), *p* < 0.001], and shorter LOS in the hospital (days) [19 (16, 24) vs. 22 (18, 27), *p* = 0.001]. No patient developed vascular access complications under anticoagulation therapy. One patient developed postoperative stroke caused by atherosclerotic plaques detached from the left subclavian artery in selective LIMA angiography. For in-hospital death, one patient died of cardiogenic shock caused by postoperative MI, the other patient died of severe lung infection in the HCR group, and one patient died of stroke in the OPCAB group.

**Table 4:** In-hospital outcomes of matched patients who underwent HCR and OPCAB.

**Variables**	**HCR group**,	**OPCAB group**,	***P***
	***N =* 151**	***N =* 151**	
In-hospital death	2 (1.3)	1 (0.7)	1.00
MI	3 (2.0)	4 (2.6)	1.00
Stroke	3 (2.0)	2 (1.3)	1.00
Repeat revascularization	0 (0.0)	1 (0.7)	1.00
Reoperation for bleeding	6 (4.0)	1 (0.7)	0.12
Time of mechanic ventilation (h)^*^	15 (16, 17)	17 (16,20)	<0.001
PMV (≥ 48 h)	17 (11.3)	19 (12.6)	0.72
Perioperative transfusion	36 (23.8)	80 (53.0)	<0.001
Renal failure needs dialysis	2 (1.3)	1 (0.7)	1.00
New onset AF	8 (5.3)	23 (15.2)	0.004
Incision infection	2 (1.3)	2 (1.3)	1.00
ICU stay (h)^*^	77 (66, 124)	95 (69, 140)	0.17
LOS in hospital (day)^*^	19 (16, 24)	22 (18, 27)	0.001

* *Non-normal variables are presented as median (P25, P75)*.

*Categorical values are presented as n (%)*.

*HCR, hybrid coronary revascularization; OPCAB, off pump coronary artery bypass grafting; MI, myocardial infarction; PMV, prolonged mechanic ventilation; AF, atrial fibrillation; ICU, intensive care unit; LOS, length of stay*.

### Midterm Outcomes

**Figure 2:**
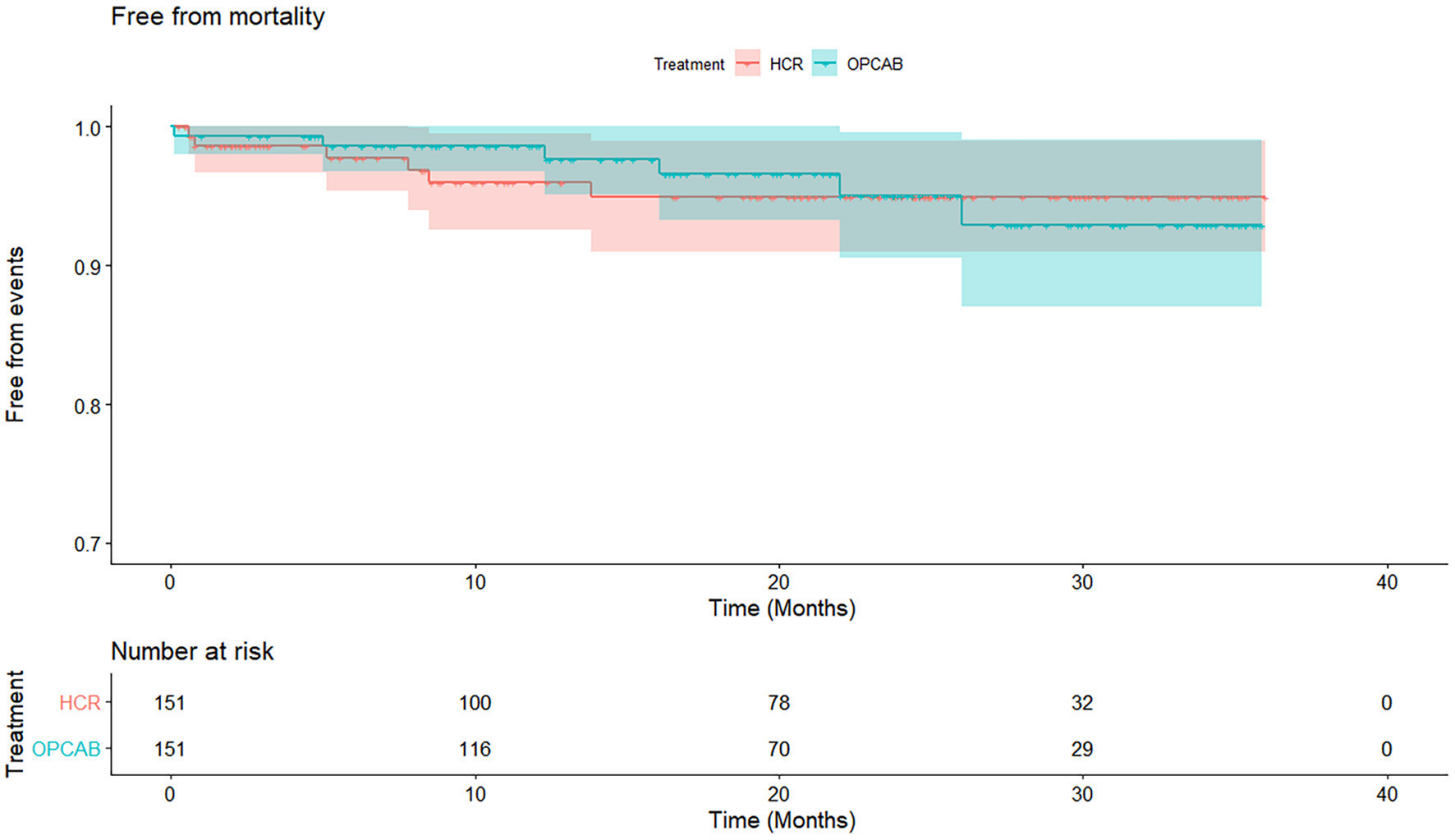
Cumulative survival rate in hybrid coronary revascularization (HCR) and off-pump coronary artery bypass (OPCAB) group. Kaplan–Meier curve estimates similar cumulated survival rate in HCR and OPCAB groups (94.9 vs. 92.9%, *p* = 0.91) during the follow-up. HCR, hybrid coronary revascularization; OPCAB, off-pump coronary artery bypass graft.

**Figure 3:**
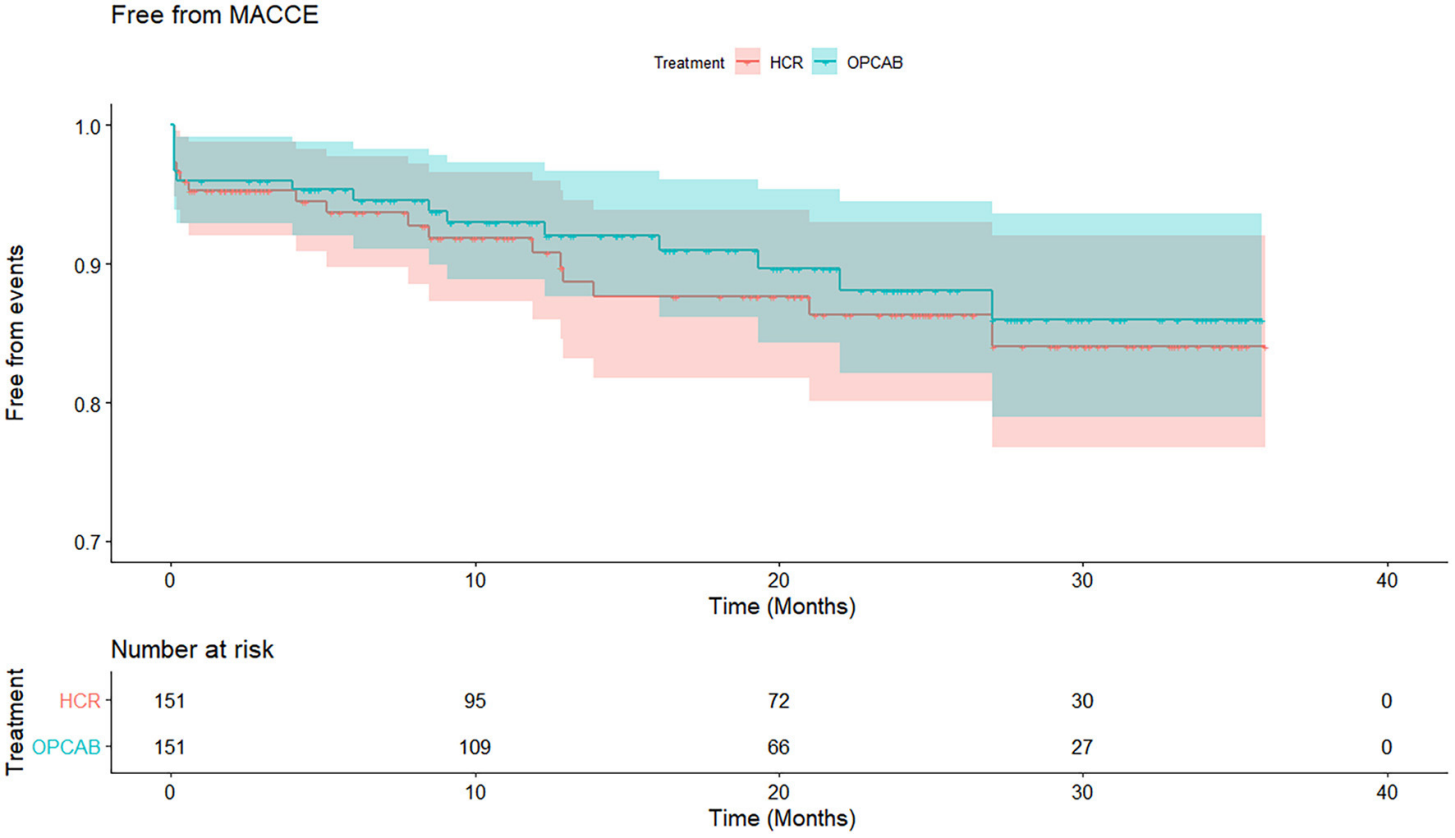
Cumulative free from major adverse cardiovascular and cerebrovascular events (MACCE) rate in HCR and OPCAB group. Kaplan–Meier curve estimates similar cumulated free from MACCE rate in HCR and OPCAB groups (84.1 vs. 86%, *p* = 0.59) during the follow-up. MACCE, major adverse cardiovascular and cerebrovascular events; HCR, hybrid coronary revascularization; OPCAB, off-pump coronary artery bypass grafting.

At a median follow-up time of 20 months (interquartile range: 10–30 months), the cumulative mortality in the HCR and OPCAB groups was 5.1 and 7.1%, respectively (log rank *p* = 0.91) (Table 5, Figure 2). Significant differences in the estimated rates of MI (4.3 vs. 4.3%, *p* = 0.56), stroke (4.0 vs. 4.5*%, p* = 0.66), repeat revascularization (4.7 vs. 5.0%, *p* = 0.61), and MACCE (15.9 vs. 14.0%, *p* = 0.59) were not observed for the HCR and CABG groups (Table 5, Figure 3).

**Table 5:** Midterm outcomes of matched patients who underwent HCR and OPCAB.

**Variables**	**HCR group**,	**OPCAB group**,	**HR (95% CI)**	***P***
	***N =* 151**	***N =* 151**		
Death	6 (5.1)	6 (7.1)	1.07 (0.34–3.30)	0.91
MI	4 (4.3)	6 (4.3)	0.69 (0.20–2.38)	0.56
Stroke	5 (4.0)	4 (4.5)	1.34 (0.36–4.95)	0.66
Repeat revascularization	4 (4.7)	6 (5.0)	0.72 (0.21–2.49)	0.61
MACCE	17 (15.9)	15 (14.0)	1.21 (0.60–2.41)	0.59

*Events are presented as n (cumulative incidence rate%) after procedure*.

*HCR, hybrid coronary revascularization; OPCAB, off pump coronary artery bypass grafting; HR, hazard ratio; CI, confidence interval; MI, myocardial infarction; MACCE, major adverse cardiac and cerebrovascular events*.

## Discussion

Minimally invasive techniques for surgical myocardial revascularization have received much attention in recent years, especially the HCR technique (17). Compared with staged HCR, one-stop HCR can achieve complete revascularization in a single procedure, which avoids ischemic events during the waiting period caused by incomplete revascularization in staged HCR. Furthermore, LIMA-LAD anastomosis can be evaluated by angiography immediately before PCI, and grafts can be revised if there are any major issues (18). Additionally, high-risk non-LAD PCI is performed with a protected LAD territory, and surgical bailout can be performed for any possible complication or unsuccessful PCI with a surgical team in a hybrid suite. Finally, this single-step procedure reduces hospital stay and readmission, provides convenience for patients, and improves patient satisfaction (19).

On the other hand, the simultaneous procedure requires a costly hybrid room featuring advanced surgical and interventional equipment. In addition, adopting an appropriate antiplatelet therapy strategy to balance the risk of bleeding and stent thrombosis is a major challenge (20).

To date, a series of published data comparing HCR with conventional CABG and OPCAB from different centers with variable surgical techniques and study methodologies have demonstrated limited conclusions (11, 12, 21, 22). Most of the patients involved in these studies underwent staged HCR, and the one-stop HCR approach accounted for only 15% of all HCR procedures in the United States (23). Outcomes of one-stop HCR should be proven, particularly due to their different natural attributes.

The present study compared early and midterm results between one-stop HCR and standard OPCAB, which revealed similar excellent in-hospital and midterm outcomes. In addition, the HCR group was associated with a lower rate of perioperative transfusion (23.8 vs. 53.0%), new-onset AF (5.3 vs. 15.2%), shorter time of mechanical ventilation, and LOS in the hospital. Consistent with our results, Reynolds et al. (24) evaluated a total of 4,260 patients (1,350 of whom underwent HCR) in a meta-analysis. They confirmed that HCR had a significantly lower rate of blood transfusion than CABG (22.8 vs. 46.1%) and a shorter time of mechanical ventilation and LOS in the hospital, but no significant differences were found in ICU stay, postoperative atrial fibrillation, renal failure, perioperative myocardial infarction, or death. Similar conclusions were also investigated by Sardar et al. (25). These findings indicated the advantages of minimally invasive and rapid recovery of HCR.

Another particular potential benefit of HCR procedures is completely avoiding manipulation of the aorta, which could theoretically reduce the risk of neurological events. However, in our study, the incidence of in-hospital stroke was low and was comparable between the two groups. Three (HCR group) and two (OPCAB group) patients developed stroke. Among the stroke patients in the HCR group, two patients had a history of cerebral infarction, and one stroke patient was caused by atherosclerotic plaques detached from the left subclavian artery in selective LIMA angiography. Meanwhile, in the OPCAB group, using proximal anastomosis devices (Heartstring or Enclose) was a routine procedure in our center. This surgical approach reduced aortic manipulation and was associated with a lower risk of perioperative stroke (26). Nevertheless, HCR is still an optimal strategy for patients with severe aortic atherosclerosis.

Appropriate antiplatelet therapy to preserve stent patency and minimize the risk of postoperative bleeding is challenging in one-stop HCR procedures. Exposure to potent antiplatelet drugs may increase the risk of postoperative bleeding events. Coincidentally, in the present study, the reoperation rate for bleeding was higher in the HCR group (4.0 vs.0.7%), although the difference was not significant (*p* = 0.12), which is consistent with the rate of 3 to 6.8% reported by Zhao et al. and Harskamp et al. (18). Most of the reoperation cases occurred early after introducing one-stop HCR into our center, and the LIMA pedicle was harvested through direct vison at that time. Then, endoscopy was used in LIMA harvesting, which could reduce the trauma of the LIMA bed. We strengthened surgical hemostasis, such as by using the “LIMA bed closure” technique. As a consequence, few patients now develop major bleeding events requiring reoperation.

During midterm follow-up, we revealed no differences between the two groups in cumulative survival (94.9 vs. 92.9%, *p* = 0.91) or free from MACCE (84.1 vs. 86%, *p* = 0.59) after the procedure. These findings are consistent with recently published data. The HYBRID (POL-MIDES) trial (11), the largest prospective randomized study comparing HCR with conventional CABG, involved 98 HCR patients and 102 conventional CABG patients. The 5-year survival rates were 93.6% in the HCR group and 90.8% in the CABG group (*p* = 0.69), and the rates of freedom from MACCE were 45.2 and 53.4%, respectively (*p* = 0.39). No differences were found between the two groups. There were also no differences in the rates of MI, stroke, or repeat revascularization. Shen et al. (27) compared one-stop HCR, CABG, and PCI in an observational study. At the 3-year follow-up, actuarial survival was 99.3% in the HCR group and 97.2% in the CABG group, and the cumulative rate of freedom from MACCE was 93.6% after HCR and 86.5% after CABG (*p* = 0.14). In Shen's study, surgical revascularization was completed through a lower partial ministernotomy, which is different from the widely used technique through a small anterior thoracotomy.

In the present study, the rate of any repeat revascularization was comparable between the HCR and OPCAB groups (4.7 vs. 5.0%; OR.72; 95% CI.21–2.49; *p* = 0.61) during the follow-up. Hage et al. (12) compared HCR (*n* = 147, robotic-assisted minimally invasive direct CABG) and OPCAB (*n* = 216) using inverse-probability weighting. The HCR was associated with a higher in-hospital reintervention rate (HCR 3.4% vs. CABG 0%, *p* = 0.03). For long-term follow-up, freedom from any form of revascularization was similar between the two groups (91 vs. 92%; *p* = 0.80), which is consistent with our results. In contrast, a meta-analysis from Nolan et al. (28) found that HCR was also associated with a higher risk of long-term repeat target vessel revascularization (TVR) than CABG.

Several limitations need to be addressed. First, this study was a single-center retrospective study, and the risk of selection bias was inevitable despite the benefits of propensity score matching. Second, the sample size was limited. Finally, the follow-up time was short, and long-term follow-up to verify the effectiveness of one-stop HCR is warranted.

## Conclusions

One-stop HCR is safe and efficacious with less invasiveness and faster postoperative recovery in selected patients with multivessel coronary artery disease. Compared with OPCAB, one-stop HCR is associated with a lower rate of perioperative transfusion and new-onset AF, shorter time of mechanical ventilation and LOS in the hospital, and excellent similar midterm outcomes. Randomized controlled trials with larger sample sizes and long-term follow-up are warranted to confirm these findings.

## Data Availability Statement

The original contributions presented in the study are included in the article/supplementary material, further inquiries can be directed to the corresponding authors.

## Ethics Statement

The studies involving human participants were reviewed and approved by Research Ethics Board of Chaoyang Hospital. Written informed consent for participation was not required for this study in accordance with the national legislation and the institutional requirements.

## Author Contributions

DL, PS, and JG: conception and design. YL, JZ, and PS: administrative support. DL, JG, YGu, XA, and XZ: provision of study materials or patients. DL and YGa: collection and assembly of data. DL, JG, and SG: data analysis and interpretation. DL: manuscript writing. All authors read and approved the final manuscript.

## Funding

This work was supported by Beijing Municipal Science and Technology Commission Foundation (No. Z191100006619036).

## Conflict of Interest

The authors declare that the research was conducted in the absence of any commercial or financial relationships that could be construed as a potential conflict of interest.

## Publisher's Note

All claims expressed in this article are solely those of the authors and do not necessarily represent those of their affiliated organizations, or those of the publisher, the editors and the reviewers. Any product that may be evaluated in this article, or claim that may be made by its manufacturer, is not guaranteed or endorsed by the publisher.
